# Management of Progressive Mandibular Fibrous Dysplasia With Radical Resection and Immediate Reconstruction Using Autoclaved Autograft: A Case Report

**DOI:** 10.7759/cureus.94096

**Published:** 2025-10-08

**Authors:** Sharanbasappa Japatti, Abhijeet Lande, Sarika S Lasinkar, Yash N Nawali, Shubham D Patade

**Affiliations:** 1 Department of Oral and Maxillofacial Surgery, Jawahar Medical Foundation's Annasaheb Chudaman Patil Memorial Dental College, Dhule, IND

**Keywords:** adult, fibrous dysplasia, mandible, reconstruction, surgical

## Abstract

Fibrous dysplasia is an uncommon condition in which fibrous elements and immature bones progressively replace normal skeletal tissue. When the mandible is involved, patients may develop swelling, asymmetry, and functional difficulties, which require careful differentiation from other jaw pathologies to avoid misdiagnosis. This report describes the case of a 48-year-old man who presented with a slowly enlarging, painless swelling of the lower jaw accompanied by mobile teeth and impaired chewing ability. Clinical and radiographic investigations revealed a diffuse lesion with a characteristic ground-glass radiographic appearance, and histopathological analysis confirmed the diagnosis of fibrous dysplasia. Routine blood test results were within normal limits. Surgical management was performed using an extraoral approach. The affected bone was recontoured after osteotomy, sterilized by autoclaving, and repositioned with the aid of a reconstruction plate to maintain the mandibular form and stability. Postoperatively, healing was initially favorable; however, delayed intraoral wound breakdown with bone exposure occurred six months after the procedure. This complication was successfully corrected using a secondary procedure involving plate removal and careful closure. At one year, the patient exhibited satisfactory bone stability, improved masticatory efficiency, and acceptable esthetics without evidence of lesion progression. This case highlights the importance of accurate diagnosis, personalized surgical planning, and ongoing patient follow-up for those with mandibular fibrous dysplasia.

## Introduction

Fibrous dysplasia is a rare non-neoplastic bone disorder characterized by the replacement of normal bone with fibrous tissue and immature woven bone, leading to structural deformities and functional impairment [1]. Primarily affecting the skeletal system, it can involve a single bone (monostotic) or multiple bones (polyostotic), with the mandible being a common site of craniofacial involvement [2]. The condition often presents as painless swelling, which may cause cosmetic concerns or functional issues, such as dental malalignment or chewing difficulties, necessitating careful evaluation to differentiate it from other mandibular pathologies, including benign tumors and malignancies [2,3].

Diagnosis relies on a combination of clinical assessment, radiographic imaging, and histopathological analysis, as fibrous dysplasia shares features with other fibro-osseous lesions, making accurate differentiation critical [4]. Management is complex and often requires surgical intervention to address deformities or functional deficits while balancing the need for complete lesion management with the preservation of anatomical integrity [2]. Postoperative care is essential because of the potential complications, such as delayed healing or recurrence of symptoms. The variable presentation and potential for associated syndromes, such as McCune-Albright syndrome, add complexity to its management [5]. This case report describes the diagnostic and therapeutic journey of a patient with mandibular fibrous dysplasia, highlighting the importance of a multidisciplinary approach that involves oral surgery, radiology, and pathology. By documenting the clinical presentation, diagnostic process, and management strategies, this report aims to contribute to the understanding of fibrous dysplasia, emphasizing the importance of tailored interventions and long-term monitoring to optimize patient outcomes for this challenging condition.

## Case presentation

**Figure 1:**
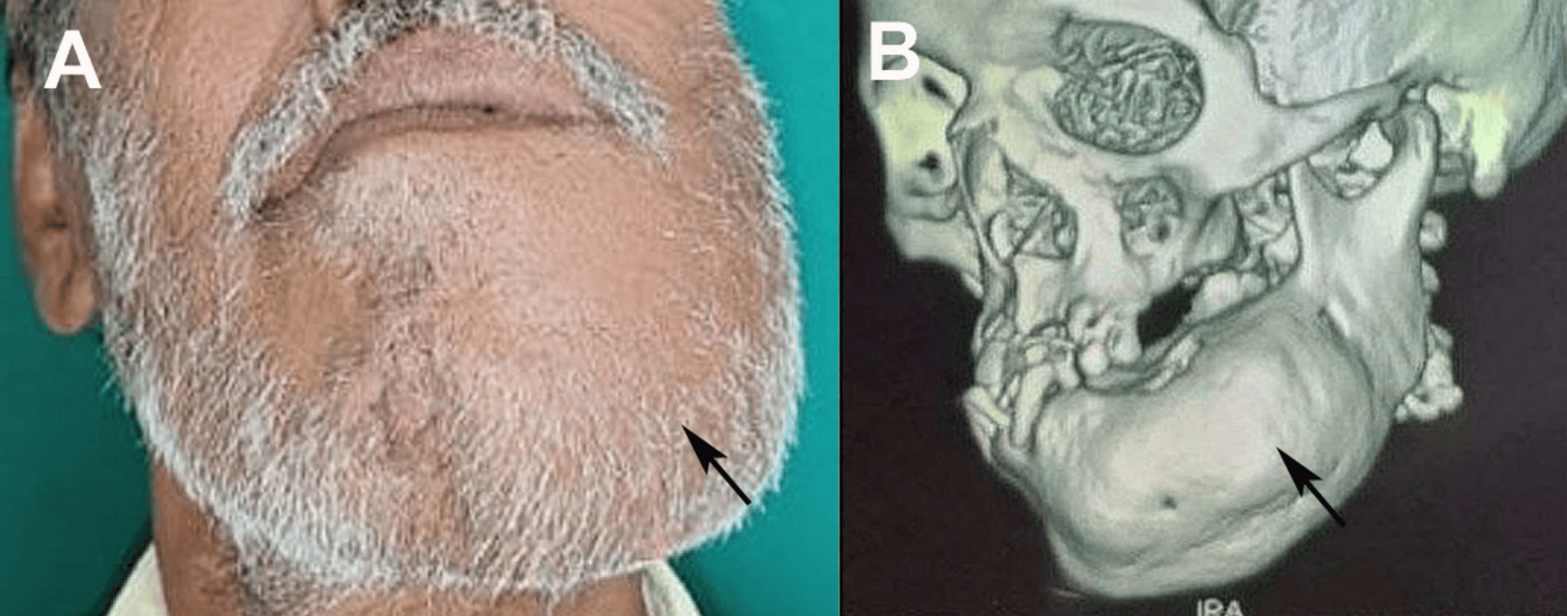
(A) Extraoral swelling on the left side with marked facial asymmetry and (B) CT showing expansion of mandibular bone without perforation on the left side Original images of the patient were used with the patient's consent for publication. CT: computed tomography

A 48-year-old man presented to the Department of Oral and Maxillofacial Surgery, Jawahar Medical Foundation’s Annasaheb Chudaman Patil Memorial Dental College, Dhule, India, in January 2024, with a chief complaint of a progressively enlarging, painless, expansile swelling affecting the left mandibular body and symphysis region, which had evolved over several months. The associated clinical findings included significant mobility of the adjacent teeth and a notable decline in periodontal support, resulting in functional impairment during mastication and challenges in maintaining oral hygiene. The medical, familial, and trauma histories were non-contributory, with no reported constitutional symptoms, cutaneous pigmentation, or features suggestive of syndromic associations. Extraoral inspection revealed facial asymmetry, with facial swelling measuring 5 × 4 cm on the left side. In contrast, the intraoral examination revealed a firm, non-tender mass fixed to the underlying bone, with intact overlying mucosa and no cervical lymphadenopathy (Figure 1A). The clinical presentation raised suspicion of a benign fibro-osseous process; however, low-grade malignant neoplasms, such as osteosarcoma, were considered less likely differentials and therefore required exclusion. Radiographic evaluation commenced with an orthopantomogram, which illustrated a poorly demarcated radiopaque lesion crossing the mandibular midline and exhibiting pathognomonic "ground-glass" opacity. This finding supported a provisional diagnosis of fibrous dysplasia. Still, it necessitated distinction from other fibro-osseous entities, notably ossifying fibroma, which typically presents as a well-circumscribed lesion often surrounded by a radiolucent rim, and chronic sclerosing osteomyelitis, which may display layered periosteal reactions and sequestrum formation. To further characterize the anatomical extent of the lesion and its relationship with the mandibular canal and dentition, a high-resolution 3D CT scan was performed, which corroborated the ill-defined, expansile, and radiodense nature of the lesion (Figure 1B). An incisional biopsy was performed under local anesthesia for histopathological analysis.

**Figure 2:**
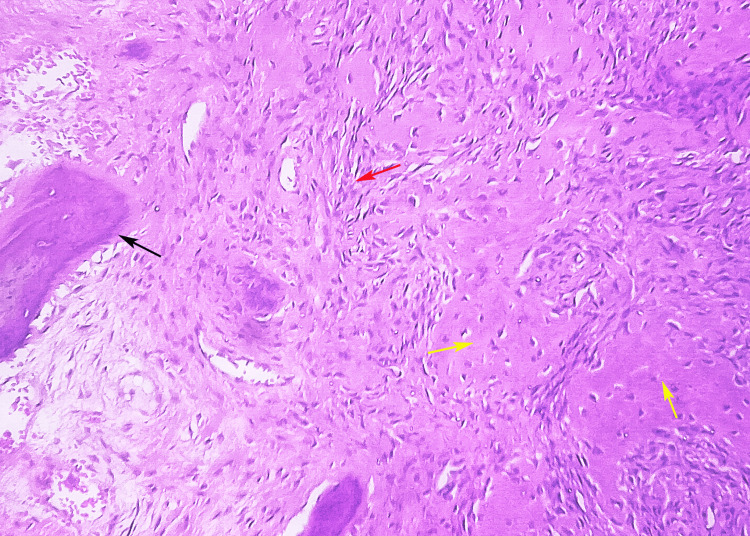
Hematoxylin and eosin-stained section at 40X magnification showed peripheral host bone (black arrow), osteoid formation (yellow arrow), and a fibrous component (red arrow) Original image of histopathological section of lesion at 40X magnification stained with hematoxylin and eosin stain.

Microscopic examination revealed a moderately cellular fibroblastic stroma harboring irregular, curvilinear trabeculae of immature woven bone that characteristically lacked osteoblastic rimming, conforming to the classic "Chinese character" morphology, confirming the diagnosis of fibrous dysplasia. This histologic architecture effectively excludes ossifying fibroma, which demonstrates osteoblastic rimming and more organized bone formation, and osteosarcoma, which exhibits significant cellular atypia, aberrant mitotic figures, and malignant osteoid production (Figure 2). Serologic analysis, including a complete blood count, serum calcium, and alkaline phosphatase levels, yielded results within normal reference ranges, thereby ruling out metabolic bone disorders, such as hyperparathyroidism, as a consideration. The aggregate clinical, radiologic, and histopathologic evidence culminated in a definitive diagnosis of monostotic fibrous dysplasia of the mandible.

**Figure 3:**
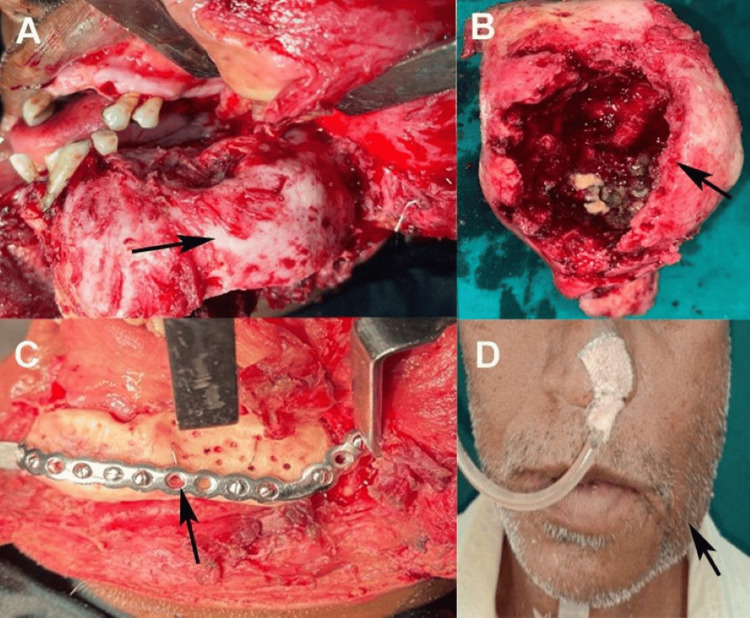
(A) Surgical exposure of a 5 x 4 cm mandibular fibrous dysplasia via submandibular incision with lateral lower lip split, (B) excised lesion prior to reshaping, (C) readaptation of autoclaved, reshaped mandibular bone fixed with a reconstruction plate, and (D) extraoral view demonstrating satisfactory postoperative healing at six weeks Original images of the patient were used with the patient's consent for publication.

Surgical management was planned to address the functional and esthetic effects of the lesions. The procedure was performed under general anesthesia using an extraoral approach via a submandibular incision combined with a lateral lower lip split to ensure adequate surgical exposure (Figure 3A). The lesion was osteotomized with safe margins to reduce bulk and prevent further expansion while preserving as much healthy bone as possible. The excised specimen underwent thorough removal of all soft tissues, followed by cleansing with saline and betadine solution. The enlarged segment of the structure was reshaped. Subsequently, this specimen was subjected to autoclaving at 121°C under 15 psi for 40 minutes. Numerous perforations were created in the autoclaved bone, after which the specimen was rinsed with sterile saline and affixed to the remaining mandible utilizing a reconstruction plate and screws, following intermaxillary fixation and verification of the condylar position within the glenoid fossa (Figure 3B-3C). Closure was performed in layers, both intraorally and extraorally, to promote optimal wound healing and minimize complications.

Postoperative care involved regular follow-up to monitor healing and assess complications. Extraoral healing was satisfactory, with well-healed incision sites and no signs of infection or dehiscence (Figure 3D). However, intraoral healing was delayed, likely due to the surgical complexity, compromised periodontal status, and tension during intraoral closure. At the six-month follow-up, a complication was noted: intraoral exposure of the reconstructed bone, which posed a risk of infection or further bone compromise. A secondary procedure was performed under local anesthesia, involving the removal of the reconstruction plate, minimal trimming of the exposed bone to smooth the irregular edges, and meticulous reclosure of the intraoral wound. The patient was prescribed antibiotics, and detailed instructions for oral hygiene were provided to promote healing and prevent infection.

Following the secondary intervention, healing progressed satisfactorily, with resolution of bone exposure and no further wound breakdown noted. The patient was monitored over the subsequent six months with regular clinical examinations and periodic radiographic assessments to evaluate bone stability and monitor disease progression, as fibrous dysplasia can exhibit slow growth. At the latest follow-up, no signs of lesion expansion or additional complications were noted. The patient reported improved oral function, including better chewing ability, and was satisfied with the aesthetic outcome, as the mandibular contour was well-preserved. Periodontal health is stabilized through ongoing dental care, including professional cleaning and patient education.

## Discussion

Fibrous dysplasia of the mandible, a benign fibro-osseous lesion characterized by the replacement of normal bone with fibrous tissue and immature woven bone, typically presents as painless, slowly progressive swelling, often in younger patients [1]. It constitutes approximately 2.5% of all osseous lesions and 7% of all benign osseous neoplasms [6]. Because numerous patients remain asymptomatic and are incidentally identified following oral radiographic examinations, accurately assessing the incidence of fibrous dysplasia presents considerable challenges [7]. The asymptomatic nature of fibrous dysplasia may explain the extensive growth observed in our patient. Most cases are found in the posterior mandible and are monostotic, as in our case [8].

Our patient presented with a large (5 × 4 cm) painless left mandibular swelling, associated with tooth mobility and periodontal compromise. A similar case was reported in a 24-year-old woman, with involvement of the lower right posterior region. The patient was diagnosed with monostotic fibrous dysplasia measuring 2 × 3 cm. For instance, mandibular fibrous dysplasia is reported to cause unilateral, painless enlargement, resulting in facial asymmetry, as confirmed via orthopantomogram and CT, which show a "ground-glass" opacity, similar to our findings [3,8,9]. Alawadhi et al. [10] reported a case of right mandibular fibrous dysplasia in an 18-year-old female patient who had been misdiagnosed with chronic parotitis for seven years. The hallmark “ground-glass” appearance of fibrous dysplasia, with poorly defined borders, was confirmed on panoramic radiography and 3D CT, supporting our diagnosis. This highlights the importance of early and accurate diagnosis in cases of large fibrous dysplasia.

Management strategies highlight this variability in the literature. While conservative observation is sufficient for asymptomatic or quiescent lesions, our case necessitated intervention due to progressive enlargement and periodontal issues [2,11]. We employed a radical osteotomy with autoclaved autograft and reconstruction plate fixation [7,12]. Our autoclaving method for sterilization innovatively repurposes excised bone, potentially reducing donor site morbidity compared with allografts. Hande et al. [13] reported five cases of fibrous dysplasia that were treated with surgical resection.

The complications in our case included delayed intraoral healing with bone exposure at six months, resolved by plate removal and trimming, which resonated with the reported postoperative issues. Complications associated with the management of fibrous dysplasia include bone resorption, infections, and wound dehiscence, often linked to graft volume and oral contamination, similar to our intraoral challenges, despite satisfactory extraoral healing [1,3,5]. The complication in our case highlights oral site vulnerabilities and underscores the need for enhanced hygiene protocols.

Posnick [14] advocated ongoing lifelong monitoring of fibrous dysplasia. Recurrence of fibrous dysplasia is rare in adults; however, it is more frequently observed in adolescence. Alvares et al. [15] followed a case of monostotic fibrous dysplasia for 23 years and noted spontaneous bone remodeling. De Melo et al. [16] found no recurrence during regular follow-up for four years in a 15-year-old girl with a large monostotic fibrous dysplasia of the right mandible treated with surgical resection. This finding emphasizes the importance of regular long-term follow-up in patients with fibrous dysplasia. Long-term monitoring is essential for detecting potential progression or rare malignant transformations.

The use of autoclaved autogenous bone grafts in mandibular reconstruction for fibrous dysplasia offers significant advantages, including eradication of pathological cells through sterilization, preservation of native bone contour, and elimination of donor site morbidity. This approach ensures structural stability and promotes healing through vascular ingrowth, avoiding immunologic risks associated with allografts. Previous studies confirm the efficacy of autoclaving in eliminating tumor cells while maintaining mechanical properties, supporting its reliability for immediate reconstruction [17-19]. Osawa et al. [20] revealed in their experiments that deep-freezing and autoclaving had only minimal effects on bone structure, although osteocytes were degenerated.

## Conclusions

Mandibular fibrous dysplasia presents unique diagnostic and therapeutic challenges, often mimicking other fibro-osseous lesions. Accurate diagnosis through radiographic and histopathological evaluations is essential for guiding management. Our case highlights the role of surgical intervention in addressing functional and esthetic concerns with the innovative use of autoclaved autografts to ensure mandibular continuity. Despite postoperative complications such as intraoral bone exposure, timely secondary management and vigilant follow-up achieved favorable outcomes. This report emphasizes the importance of a multidisciplinary approach, meticulous surgical planning, and long-term monitoring to optimize patient function, esthetics, and quality of life in patients with fibrous dysplasia.

## References

[1] Kruse A, Pieles U, Riener MO, Zunker Ch, Bredell MG, Grätz KW (2009). Craniomaxillofacial fibrous dysplasia: a 10-year database 1996-2006. Br J Oral Maxillofac Surg.

[2] Macdonald-Jankowski DS, Li TK (2009). Fibrous dysplasia in a Hong Kong community: the clinical and radiological features and outcomes of treatment. Dentomaxillofac Radiol.

[3] Gupta D, Garg P, Mittal A (2017). Computed tomography in craniofacial fibrous dysplasia: a case series with review of literature and classification update. Open Dent J.

[4] Kushchayeva YS, Kushchayev SV, Glushko TY, Tella SH, Teytelboym OM, Collins MT, Boyce AM (2018). Fibrous dysplasia for radiologists: beyond ground glass bone matrix. Insights Imaging.

[5] Hartley I, Zhadina M, Collins MT, Boyce AM (2019). Fibrous dysplasia of bone and McCune-Albright syndrome: a bench to bedside review. Calcif Tissue Int.

[6] Menon S, Venkatswamy S, Ramu V, Banu K, Ehtaih S, Kashyap VM (2013). Craniofacial fibrous dysplasia: surgery and literature review. Ann Maxillofac Surg.

[7] Pacino GA, Cocuzza S, Tonoli G (2020). Jawbone fibrous dysplasia: retrospective evaluation in a cases series surgically treated and short review of the literature. Acta Biomed.

[8] Worawongvasu R, Songkampol K (2010). Fibro-osseous lesions of the jaws: an analysis of 122 cases in Thailand. J Oral Pathol Med.

[9] Assiri KI (2020). Monostotic fibrous dysplasia involving the mandible: a case report. SAGE Open Med Case Rep.

[10] Alawadhi R, Alsairefi S, AlMutairi MM (2025). Misdiagnosis of a paediatric fibrous dysplasia of the mandible. Cureus.

[11] Kreutziger KL (1989). Giant fibrous dysplasia of the mandible: surgical management. Laryngoscope.

[12] Lee JS, FitzGibbon EJ, Chen YR (2012). Clinical guidelines for the management of craniofacial fibrous dysplasia. Orphanet J Rare Dis.

[13] Hande A, Kalmegh P, Patil S, Sonone A, Pakhale A (2024). Monostotic fibrous dysplasia of jaw bones: a case series. BMC Oral Health.

[14] Posnick JC (1998). Fibrous dysplasia of the craniomaxillofacial region: current clinical perspectives. Br J Oral Maxillofac Surg.

[15] Alvares LC, Capelozza AL, Cardoso CL, Lima MC, Fleury RN, Damante JH (2009). Monostotic fibrous dysplasia: a 23-year follow-up of a patient with spontaneous bone remodeling. Oral Surg Oral Med Oral Pathol Oral Radiol Endod.

[16] De Melo WM, Sonoda CK, Hochuli-Vieira E (2012). Monostotic fibrous dysplasia of the mandible. J Craniofac Surg.

[17] Lee JW, Tsai SS, Kuo YL (2006). Transient recycling of resected bone to facilitate mandibular reconstruction--a technical note. J Craniomaxillofac Surg.

[18] Singh VA, Nagalingam J, Saad M, Pailoor J (2010). Which is the best method of sterilization of tumour bone for reimplantation? A biomechanical and histopathological study. Biomed Eng Online.

[19] Menon S, Banu K, Veena R, Srihari V, Sham ME, Vinay M (2015). The recycling of autoclaved autografts in mandibular reconstruction: case report and review of literature. J Maxillofac Oral Surg.

[20] Osawa M, Hara H, Ichinose Y, Koyama T, Kobayashi S, Sugita Y (1990). Cranioplasty with a frozen and autoclaved bone flap. Acta Neurochir (Wien).

